# Interface Structures and Electronic States of Epitaxial Tetraazanaphthacene on Single-Crystal Pentacene

**DOI:** 10.3390/ma14051088

**Published:** 2021-02-26

**Authors:** Yuki Gunjo, Hajime Kamebuchi, Ryohei Tsuruta, Masaki Iwashita, Kana Takahashi, Riku Takeuchi, Kaname Kanai, Tomoyuki Koganezawa, Kazuhiko Mase, Makoto Tadokoro, Yasuo Nakayama

**Affiliations:** 1 Department of Pure and Applied Chemistry, Graduate School of Science and Technology, Tokyo University of Science, 2641 Yamazaki, Noda, Chiba 278-8510, Japan; 7220528@ed.tus.ac.jp (Y.G.); 7218705@ed.tus.ac.jp (R.T.); 7218508@alumni.tus.ac.jp (M.I.); 7219543@ed.tus.ac.jp (K.T.); 7219545@ed.tus.ac.jp (R.T.); 2 Department of Chemistry, Faculty of Science Division I, Tokyo University of Science, 1-3 Kagurazaka, Shinjuku-ku, Tokyo 162-8601, Japan; hkame@chs.nihon-u.ac.jp (H.K.); tadokoro@rs.tus.ac.jp (M.T.); 3 Department of Physics, Faculty of Science and Technology, Tokyo University of Science, 2641 Yamazaki, Noda, Chiba 278-8510, Japan; kaname@rs.tus.ac.jp; 4 Division of Colloid and Interface Science, Research Institute for Science & Technology, Tokyo University of Science, 2641 Yamazaki, Noda, Chiba 278-8510, Japan; 5 Industrial Application Division, Japan Synchrotron Radiation Research Institute (JASRI), 1-1-1 Kouto, Sayo, Hyogo 679-5198, Japan; koganeza@spring8.or.jp; 6 Institute for Materials Structure Science, High Energy Accelerator Research Organization (KEK) and SOKENDAI, 1-1 Oho, Tsukuba, Ibaraki 305-0801, Japan; mase@post.kek.jp

**Keywords:** organic semiconductor, donor-acceptor interface, p-n junction, heteroepitaxy, grazing-incidence wide-angle X-ray scattering, photoemission

## Abstract

The structural and electronic properties of interfaces composed of donor and acceptor molecules play important roles in the development of organic opto-electronic devices. Epitaxial growth of organic semiconductor molecules offers a possibility to control the interfacial structures and to explore precise properties at the intermolecular contacts. 5,6,11,12-tetraazanaphthacene (TANC) is an acceptor molecule with a molecular structure similar to that of pentacene, a representative donor material, and thus, good compatibility with pentacene is expected. In this study, the physicochemical properties of the molecular interface between TANC and pentacene single crystal (PnSC) substrates were analyzed by atomic force microscopy, grazing-incidence X-ray diffraction (GIXD), and photoelectron spectroscopy. GIXD revealed that TANC molecules assemble into epitaxial overlayers of the (010) oriented crystallites by aligning an axis where the side edges of the molecules face each other along the $\left[\bar{1}10\right]$ direction of the PnSC. No apparent interface dipole was found, and the energy level offset between the highest occupied molecular orbitals of TANC and the PnSC was determined to be 1.75 eV, which led to a charge transfer gap width of 0.7 eV at the interface.

## 1. Introduction

Acenes are one of the most essential classes of organic semiconductor material. Not only the unsubstituted acenes themselves, but also various acene-based molecules with functional side groups and/or heterocycles have been used for prototype and practical organic semiconductor devices. The most recognized acene in the organic electronics field is the five-membered one, pentacene, which has been attracting wide attention because of its unique physicochemical properties. A significant charge carrier mobility of 35 cm^2^ V^−1^ s^−1^ at room temperature has been reported by space-charge-limited current measurements [1], and intermolecular electronic band dispersion was confirmed by angle-resolved photoelectron spectroscopy for the pentacene single crystals (PnSCs) [2,3]. In addition, pentacene is known to exhibit singlet exciton fission efficiently, which makes this molecule quite promising for application as an organic solar cell material [4,5,6].

No matter how promising the characteristics of pentacene are, they are not sufficient for realizing practical p–n junction devices such as solar cells because pentacene, and other unsubstituted acenes such as naphthacene (tetracene), are mostly p-type (donor) materials and it is not easy to switch the polarity of these molecules. To make favorable pairs with these p-type aromatic hydrocarbons, a number of complementary n-type (acceptor) compounds have been synthesized by the introduction of elements of higher electronegativities such as halogens and nitrogen to their backbones [7,8,9,10,11]. In particular, molecules with nitrogen-containing heterocycles are attracting recent interest as non-fullerene acceptor molecules for the application of organic solar cells and as high mobility n-type organic semiconductors [12]. In this context, the target material of this work, 5,6,11,12-tetraazanaphthacene (TANC), where four methine groups in naphthacene are replaced by four nitrogen atoms, is one prominent example known to exhibit the n-type semiconductor operation [7].

In this study, the structural and electronic properties of a heteroepitaxial molecular p–n heterojunction formed by stacking TANC on PnSC surfaces were investigated by non-contact mode atomic force microscopy (nc-AFM), grazing-incidence X-ray diffraction (GIXD), X-ray and ultraviolet photoelectron spectroscopy (XPS and UPS), and ultraviolet-visible absorption spectroscopy (UV-vis). Heteroepitaxy has been used as a fundamental technology for obtaining highly ordered semiconductor crystallites and can also be applied for van der Waals molecular materials [13,14]. For example, C_60_ molecules form heteroepitaxial overlayers of an in-plane mean crystallite size of over 100 nm on the single-crystal surfaces of pentacene and rubrene [15,16,17,18]. Moreover, the occurrence of energy-momentum dispersion of the intermolecular electronic bands has been observed for heteroepitaxial perfluoropentacene (PFP), which has a similar molecular shape to pentacene, stacked on the PnSC [19]. The present work revealed that TANC also grows epitaxially on the PnSC surface to form well-ordered crystalline heterojunctions. An energy level diagram at the TANC/PnSC heterojunction is determined based on the XPS and UPS results.

## 2. Materials and Methods

PnSC platelets were prepared using a physical vapor transport technique and were fixed on Au-coated (for XPS and UPS experiments [20,21]) or bare (for GIXD and AFM [15]) Si wafer pieces to prepare the “substrates”. The PnSC surface was exposed to air and ambient light after fabrication, and thus, the surface was presumed to incorporate a few percent of oxidized species [20,22]. TANC was deposited on the PnSC surfaces at room temperature in ultra-high vacuum conditions (pressure below 1 × 10^−4^ Pa) to prepare the samples. The deposition rate of TANC was set at 14–18 pm/s unless otherwise noted.

The crystallographic structure of the TANC/PnSC heterojunctions was determined by GIXD conducted at BL19B2 of SPring-8. The X-ray wavelength and glancing angle were fixed at 1.00 Å and 0.12°, respectively. Two-dimensional GIXD (2D-GIXD) images were collected by using a PILATUS-300K detector. The camera length was fixed at 172.6 mm from the rotation center of the sample azimuthal angle φ that was rotated 360° at the 0.5° interval. The details of the experimental setup are described elsewhere [15,18]. The surface morphologies of the samples were observed by nc-AFM (NaioAFM, Nanosurf, Liestal, Switzerland). The 2D-GIXD and nc-AFM measurements were carried out in atmospheric conditions (ex situ) at room temperature.

The XPS and UPS experiments were carried out at BL-13B [23] of the Photon Factory, KEK, by using a concentric hemispherical analyzer (SES-200, Gammadeta-Scienta, Uppsala, Sweden). The excitation photon energies (hν) were set at 670 eV for XPS and at 30 eV for UPS. The sample was illuminated by continuous-wave laser light (405 nm) for canceling photoemission-induced positive charges trapped in the crystal by the assistance of photoconductivity [20,24,25]. The work function of the analyzer was determined to be 4.47 eV using the Fermi edge position after careful calibration of the excitation photon energy by referring to the Fermi edge position excited by the third-order photon at the identical monochromator setting [26], and the sample work function was estimated from the minimum kinetic energy of the secondary electron region obtained with a negative bias voltage applied to the sample (*V_s_* = −5 V). The excitation energies were calibrated by using Ta4f_7/2_ peaks (with binding energy (BE) of 21.8 eV [27]) or the Fermi edge from a piece of Ta foil attached to the experimental equipment used as a reference sample, and abscissas were taken with respect to the Fermi level. For the XPS and UPS experiments, TANC was deposited in a step-by-step manner up to a thickness of 50 nm on the PnSC samples, and the XPS and UPS measurements were conducted in situ, i.e., without breaking the vacuum, throughout each experimental series, and in a normal emission geometry. The optical band gap energies of pentacene and TANC were estimated using a UV–vis spectrophotometer (UV-1800, Shimadzu, Kyoto, Japan) on 20 nm thick films of the respective materials deposited on quartz plates. All the measurements were carried out at room temperature.

## 3. Results and Discussion

### 3.1. Crystallographic Analyses

**Figure 1:**
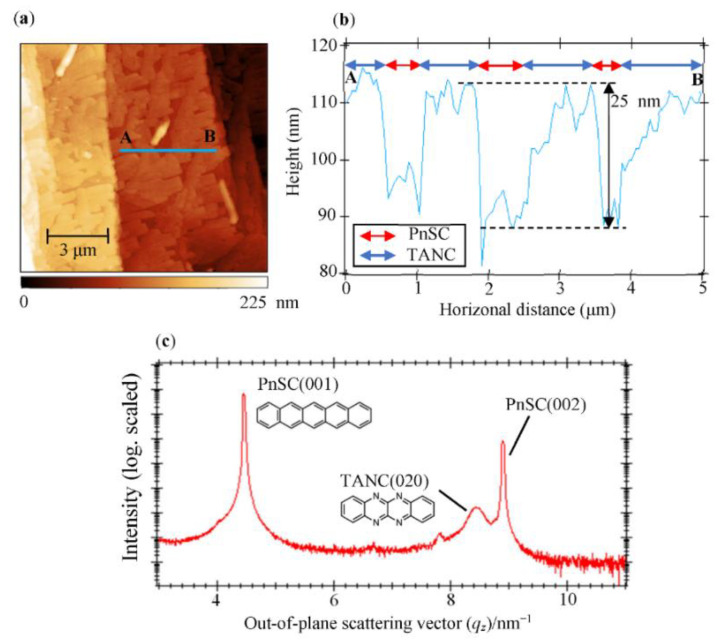
(**a**) 12.5 × 12.5 μm^2^ non-contact mode atomic force microscopy (nc-AFM) image of a pentacene single crystal (PnSC) sample with 20 nm-thick 5,6,11,12-tetraazanaphthacene (TANC). (**b**) Cross-section profile along the line A-B in (**a**). (**c**) Out-of-plane X-ray diffraction data of the TANC(20 nm)/PnSC heterojunction. Molecular structures of pentacene and TANC are also given.

An nc-AFM image of a PnSC sample with 20 nm-thick TANC is shown in Figure 1a. About 90% of the PnSC surface was covered with TANC islands of a relatively uniform height of approximately 25 nm on average, as seen in a cross-section profile across a terrace (Figure 1b). The islands were, on the whole, rectangular shaped with straight edges and of uniform size. In addition, the in-plane orientation of these islands looked regular, which implied the epitaxial growth of TANC along with a specific direction of the PnSC surface lattice [15,19,28].

Figure 1c shows out-of-plane X-ray diffraction data taken from another TANC(20 nm)/PnSC heterojunction sample. Sharp peaks at *q_z_* = 4.45 nm^−1^ and 8.90 nm^−1^ correspond to the (001) and (002) diffraction spots, respectively, of the PnSC substrate [15], whereas a broader one centered at *q_z_* = 8.45 nm^−1^ is attributable to the (020) reflection of the reported crystal structure of TANC [8]. Therefore, it can be concluded that TANC grows on the PnSC surface by aligning its b* axis perpendicular to the PnSC surface. From the TANC diffraction peak width, an out-of-plane mean crystallite size is evaluated to be 26 (±3) nm by the Scherrer equation. This value is in good agreement with the island height seen in the AFM image. It is worth noting that the TANC(020) diffraction peak width for a 50 nm-thick TANC/PnSC sample was identical to that of the 20 nm-thick sample, suggesting that the in-plane mean crystallite size was substantially independent of the TANC thickness, at least up to 50 nm-thick.

**Figure 2:**
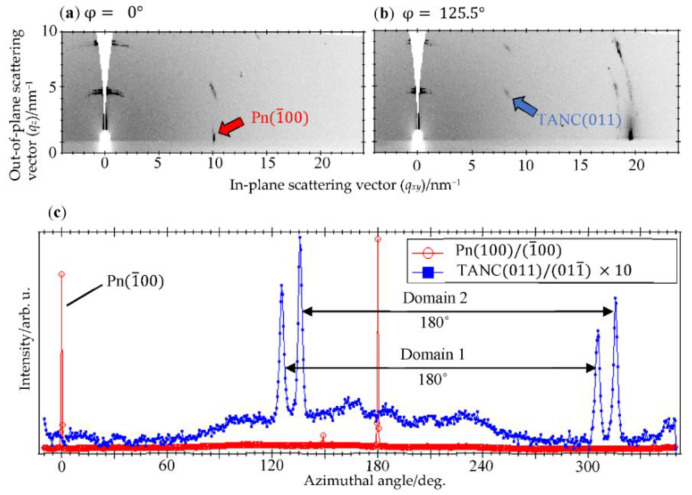
(**a**) 2D-GIXD image of the TANC(20 nm)/PnSC taken at $\varphi =0{}^{{}^{\circ}}$. (**b**) 2D-GIXD image of the same sample as in (**a**) taken at $\varphi =125.5{}^{{}^{\circ}}$. (**c**) Diffraction intensities of the PnSC$\left(100\right)/\left(\bar{1}00\right)$ and TANC $\left(011\right)/\left(01\bar{1}\right)$ spots plotted as a function of φ. The vertical scale is normalized for the maximal intensity of each curve.

**Figure 3:**
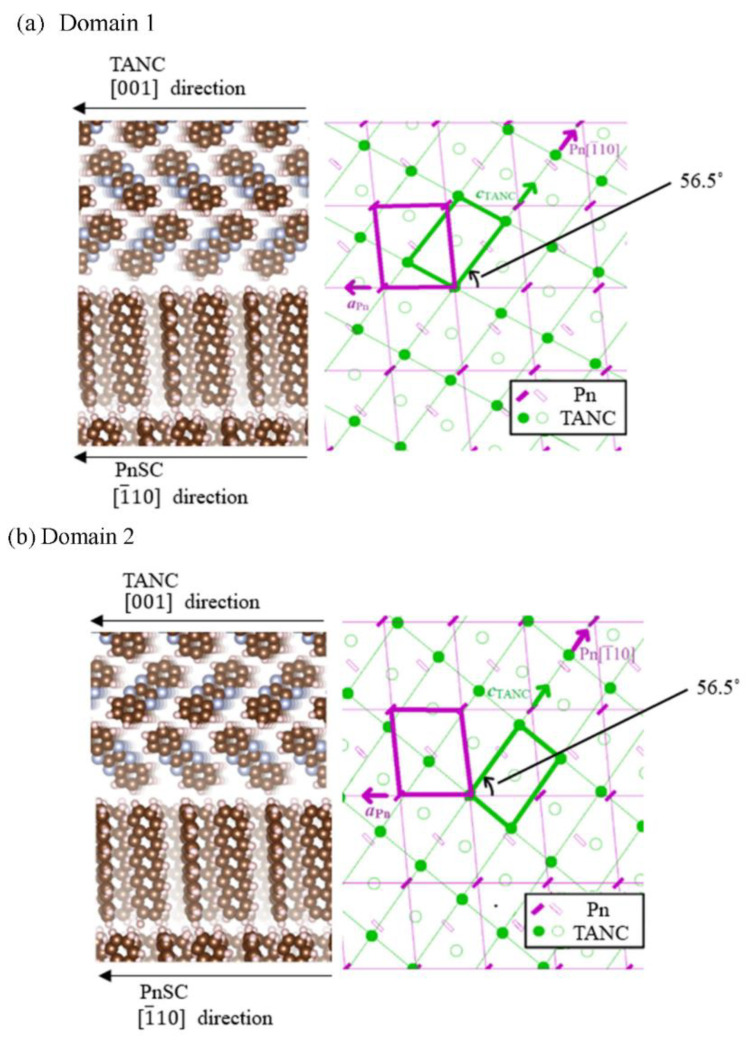
A schematic drawing of the expected molecular orientations for (**a**) Domain 1 and (**b**) Domain 2.

Figure 2a shows a 2D-GIXD image of a PnSC sample with a 20 nm-thick TANC overlayer taken at a certain azimuthal angle. In this image, a spot attributed to the $\left(\bar{1}00\right)$ diffraction of PnSC was resolved at $\left(q_{xy},\;q_{z}\right)=\left(10.1\;{\mathrm{nm}}^{-1},\;0\;{\mathrm{nm}}^{-1}\right)$. We hereafter define this sample azimuthal angle as $\varphi =0{}^{{}^{\circ}}$. In-plane rotation of the sample by $125.5{}^{{}^{\circ}}$ counterclockwise changed the 2D-GIXD pattern, as shown in Figure 2b. A spot that emerged at $\left(q_{xy},\;q_{z}\right)=\left(8.21\;{\mathrm{nm}}^{-1},\;4.24\;{\mathrm{nm}}^{-1}\right)$ cannot be assigned to any diffraction from pentacene and is attributable to the $\left(01\bar{1}\right)$ diffraction of TANC. This spot appeared only at around $\varphi =125.5{}^{{}^{\circ}},\;136{}^{{}^{\circ}},\;305.5{}^{{}^{\circ}},$ and $316{}^{{}^{\circ}}$, as shown in Figure 2c. These four orientations can be classified into two pairs with a 180˚ periodicity for both: a pair for $\varphi =125.5{}^{{}^{\circ}}$ and $305.5{}^{{}^{\circ}}$, and the other for $\varphi =-44{}^{{}^{\circ}}\;\left(=316{}^{{}^{\circ}}\right)$ and $136{}^{{}^{\circ}}$. This suggests that TANC grew epitaxially on the PnSC surface into two types of domains with different lattice orientations. We designate these two domains as “Domain 1” and “Domain 2”, as indicated in Figure 2c. The φ interval between these two pairs was $169.5{}^{{}^{\circ}}$, which corresponds to the angle difference of the (0n1) reflections for the (010) and the $\left(0\bar{1}0\right)$ surfaces ($170.60{}^{{}^{\circ}}$). Therefore, Domains 1 and 2 correspond to the “front side face” and “back side face”, respectively, of the b*-oriented TANC crystal. The surface lattice of PnSC, (001) or $\left(00\bar{1}\right)$, can be determined from the diffraction conditions of the secondary axis, namely (01n). In the present case, it is determined to be $\left(00\bar{1}\right)$. With this in mind, the interlattice relationships between the epitaxial TANC and the PnSC $\left(00\bar{1}\right)$ surface are, therefore, deduced, as shown in Figure 3a,b. In both domains, the *c*-axis of TANC, in which two adjacent TANC molecules make contact side-by-side with dual CH∙∙∙N hydrogen bonds [8], is aligned to the $\left[\bar{1}10\right]$ direction of the PnSC surface. This aligning direction is common to the cases of the epitaxial C_60_ and perfluoropentacene on PnSC [15,19].

### 3.2. Electronic Analyses

**Figure 4:**
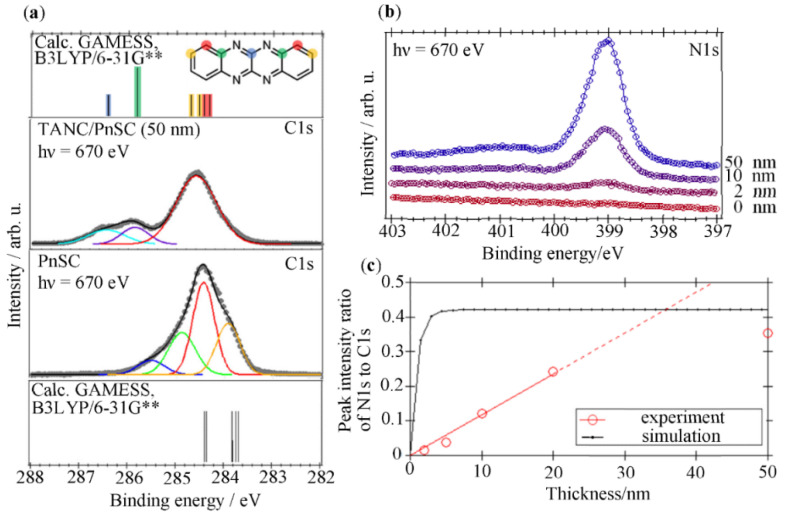
(**a**) C1s XPS spectra of the PnSC and TANC(50 nm)/PnSC interface. Quantum chemical calculation results for the C1s energy level distributions of TANC and pentacene molecules at fixed internal coordinates in the respective molecular crystals are also indicated as vertical bars, where the vertical scale is slid to align the energy position to the experimental peak position. (**b**) Evolution of N1s XPS spectra with increasing TANC thickness (2, 10, 50 nm) on PnSC. (**c**) N1s/C1s intensity ratio in this experiment (circles). A fitting line for the data points at and below 20 nm (red) and an expected ratio under the assumption of the layer-by-layer growth mode for TANC on the PnSC surface (black) are also indicated.

Figure 4a shows C1s XPS spectra of the PnSC and the TANC/PnSC samples. The C1s peak for PnSC can be separated into four components, as indicated by the red, orange, green, and blue curves. The main peak (red) and the low BE component (orange) are attributed to the carbon atoms in the “normal” pentacene molecules in the crystal, whereas the green one is attributed to the top surface carbon atoms [20,29,30]. The blue component is considered to be due to the formation of oxides on the PnSC surface [20,22]. The presence of the oxides on the PnSC surface was also confirmed by the O1s XPS results (spectra not shown). The C1s profile underwent a significant change upon the deposition of TANC: the main peak became broader and shifted to the deep BE side, and two additional components emerged at deeper BE positions. These characteristics of the C1s profile were, on the whole, reproduced by quantum chemical calculation results. While the concomitance of the deep-BE side peak was also reported in a previous XPS work for vacuum-deposited TANC on polycrystalline Au [31], it was further separated into two components attributable to four and two un-hydrogenated carbon atoms bounded with one and two nitrogen atoms, respectively, suggesting the homogeneity of the TANC molecules on the PnSC surface and/or a better energy resolution of the present experiments.

Figure 4b shows the N1s XPS spectra of the TANC/PnSC interface. The peak appeared at the 2 nm thickness, and the intensity of the peak increased as the film thickness increased. Particularly in the range of large TANC thickness, a weak structure appeared on the high BE side of the main peak. Since the difference in energy between this small feature and the main peak was close to the energy gap between the highest occupied molecular orbital (HOMO) and the lowest unoccupied molecular orbital (LUMO) of TANC (2.6 eV as discussed below), this structure is presumably attributed to the shake-up satellite as a result of the photoelectrons losing their kinetic energy through the interband transition process. Figure 4c shows the intensity ratio of the N1s peak to C1s plotted as a function of the nominal TANC thickness. The core-level peak intensity represents the abundance of the corresponding element at the sample surface, and its evolution can be modeled if an adequate growth mode of the interface is assumed. For instance, under an assumption of the layer-by-layer growth mode for TANC on the PnSC surface, the N1s/C1s intensity ratio is expected to evolve like the black curve in Figure 4c as a function of the nominal TANC thickness, where 0.12 and 0.20 Mb for the photoionization cross-sections for C1s and N1s subshells, respectively; for the 670 eV photons [32], 1.41 and 1.11 nm for the inelastic mean free paths for photoelectrons excited from the C1s and N1s states, respectively, through the solid-state (bulk) TANC [33]; 0.685 and 0.536 nm^3^ for the unit cell volume for pentacene and TANC, respectively [8,34]; and 1.491 nm for the one monolayer thickness of TANC [8] are used for this simulation. Evidently, this model does not reproduce the experimental data. Instead, the peak ratio increased proportionally to the nominal TANC thickness up to at least 20 nm. This behavior suggests Volmer-Weber growth of TANC islands that were much thicker than the mean free path of photoelectrons from the early stage of growth and the surface area occupied by the islands increased linearly to the film thickness. The extrapolation of the linear increase reached the theoretical N1s/C1s ratio at around 30 nm, and actually, the ratio for the 50 nm thick TANC saturated at that level. This suggests that TANC covered the entire surface of PnSC by that nominal thickness. It is worth noting that this assumption is also consistent with the AFM observations discussed above.

**Figure 5:**
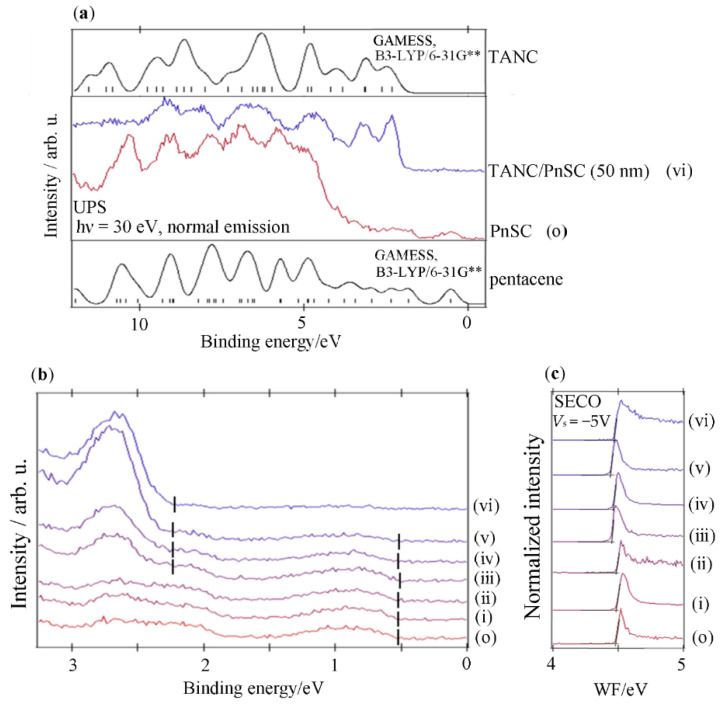
Evolution of the UPS spectra in the (**a**) wide, (**b**) valence band, and (**c**) secondary electron regions of the TANC/PnSC interfaces with increasing nominal TANC thickness as (o) 0, (i) 1, (ii) 2, (iii) 5, (iv) 10, (v) 20, and (vi) 50 nm. The DOS simulation curves obtained from the DFT calculation (GAMESS with B3LYP/6-31G** basis set) are also displayed in (**a**).

Wide-range UPS spectra of the PnSC and TANC/PnSC interface are shown in Figure 5a. Simulated density-of-states (DOS) curves of TANC and PnSC derived from the quantum chemical calculation [35] are also shown in the upper and lower curves, respectively, of Figure 5a. The calculation results reproduce the experimental spectra relatively well. Figure 5b shows the UPS spectra of the HOMO region of the PnSC and the TANC/PnSC interface. The spectral onset of pentacene HOMO was estimated to be at BE = 0.53 (±0.03) eV. This position did not change significantly until the disappearance of the PnSC-derived photoelectron signal. The HOMO-derived peaks of TANC were clearly observed at and above the thickness of 5 nm, whose onset was estimated to be at BE = 2.28 (±0.06) eV. This HOMO onset position is comparable to the previous UPS result for TANC on Au substrates [31]. From these results, the energy offset of the hole transporting levels for the TANC/PnSC interface was determined to be 1.75 (±0.09) eV. On the other hand, the work function of PnSC was 4.46 (±0.05) eV, as estimated from the secondary electron cutoff (Figure 5c), and the vacuum level shift at the TANC/PnSC interface was less than 0.05 eV. Accordingly, the ionization energy of the solid-state TANC was determined to be 6.74 (±0.11) eV.

**Figure 6:**
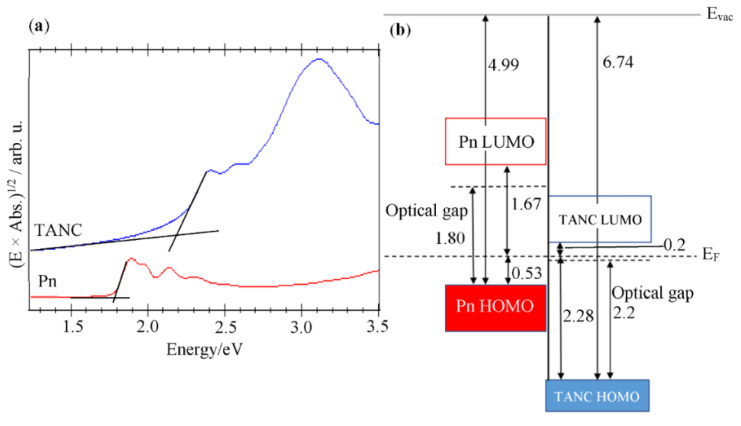
(**a**) UV–vis absorption spectra of the vacuum-deposited films of TANC and pentacene on quartz substrates. The vertical scale is taken in (energy × absorbance)^1/2^ for the estimation of the optical band gap assuming direct gap semiconductor materials. (**b**) An energy level diagram of the heterojunction between PnSC and TANC.

Whereas it is also necessary to know the LUMO positions of both pentacene and TANC to present a complete picture of the interface electronic states, UPS hardly provides any information about the unoccupied electronic states. The HOMO-LUMO gap width of solid-state pentacene has been reported to be 2.2 eV based on reliable UPS and inverse photoelectron spectroscopy results [36]. In order to estimate the HOMO-LUMO gap width of solid-state TANC, UV-vis absorption spectra (Figure 6a) were obtained on a TANC thin film formed on a quartz substrate. This revealed that the optical gap width of the solid-state TANC was 2.1 (±0.2) eV. The optical gap width of an organic semiconductor material is usually smaller than its HOMO-LUMO gap width (transport gap) because of a considerable exciton binding energy. Actually, the present UV-vis spectra for a pentacene thin film exhibited an optical gap width of 1.8 eV, which is 0.4 eV smaller than the actual HOMO-LUMO gap width. This discrepancy between these two kinds of gap widths corresponds to the exciton binding energy for the solid-state pentacene. Under a simple assumption of a common exciton binding energy for TANC to pentacene, an energy level diagram at the TANC/PnSC interface is shown in Figure 6b. As mentioned above, the vacuum level shift was negligibly small (less than 0.05 eV), and the HOMO offset between PnSC and TANC was 1.75 (±0.09) eV. In addition, the LUMO energy of TANC with respect to the Fermi level is 0.2 (±0.2) eV. Accordingly, the energy difference between the HOMO of PnSC and the LUMO of TANC was found to be 0.7 (±0.2) eV. This magnitude for the charge transfer gap width is comparable to that reported for the epitaxial heterojunction between PnSC and C_60_ (0.75 (±0.25) eV) [37]. This suggests that, whereas practical opto-electronic devices based on the heterojunctions between pentacene and TANC have not yet been reported so far, the topical p–n heterojunction could generate an open-circuit voltage of a similar range to that of known prototype organic solar cells based on C_60_/pentacene heterojunctions [38].

## 4. Conclusions

In this study, the structural and electronic properties of a p–n heterojunction of TANC molecules stacked on PnSC substrates were elucidated. 2D-GIXD demonstrated that TANC grew epitaxially in the b*–orientation and by aligning its *c*-axis along the $\left[\bar{1}10\right]$ direction of the PnSC surface where two inequivalent crystalline domains corresponding to the (010) or $\left(0\bar{1}0\right)$ facing upward were formed. An n-type character of TANC was clearly corroborated by UPS. The UPS and UV–vis results demonstrated an energy level diagram at this p-n heterojunction; the HOMO offset between PnSC and TANC was 1.75 (±0.09) eV and the charge transfer gap between the HOMO of PnSC and the LUMO of TANC was 0.7 (±0.2) eV, which is comparable to that for an epitaxial C_60_/PnSC heterojunction [37]. The concomitaance of the well-ordered molecular arrangement with strong intermolecular interactions in in-plane directions and the practical charge transfer gap width suggests the potential usefulness of the epitaxial TANC/PnSC heterojunction for, e.g., recently proposed organic solar cells with lateral-alternating donor–acceptor architectures [39] utilizing efficient transport of both positive and negative charge carriers photogenerated at the heterojunction.

## Data Availability

The data presented in this study are available in the manuscript.
